# A new deep learning model for predicting IMRT dose distributions for lung cancer with dose masks

**DOI:** 10.3389/fonc.2025.1587788

**Published:** 2025-08-19

**Authors:** Xuezhen Feng, Mingqing Wang, Xinyan Lin, Can Li, Yuxi Pan, Guoping Zuo, Ruijie Yang

**Affiliations:** ^1^ School of Nuclear Science and Technology, University of South China, Hengyang, China; ^2^ Department of Radiation Oncology, Cancer Center, Peking University Third Hospital, Beijing, China; ^3^ School of Physics, Beihang University, Beijing, China; ^4^ Institute of Operations Research and Information Engineering, Beijing University of Technology, Beijing, China

**Keywords:** deep learning, IMRT, dose prediction, radiotherapy treatment planning, lung cancer

## Abstract

**Purpose:**

3D U-Net deep neural networks are widely used for predicting radiotherapy dose distributions. However, dose prediction for lung cancer IMRT is limited to conventional radiotherapy, with significant errors in predicting the intermediate and low-dose regions.

**Methods:**

We included a mixed dataset of conventional radiotherapy and simultaneous integrated boost (SIB) radiotherapy with various prescription schemes. In addition to inputting CT images and anatomical structures, we incorporated dose mask information to provide richer local low-dose details. We trained five models with varying numbers of dose masks to investigate their impact on dose prediction models.

**Results:**

The inclusion of dose masks led to significant improvements in prediction accuracy for both the PTV and OARs. In particular, the mean absolute error (MAE) of dosimetric metrics for most OARs fell below 2%, and voxel-wise MAE within each structure steadily decreased as more dose masks were supplied—most notably in low-dose regions. These results demonstrate that incorporating dose masks effectively enhances training efficiency and prediction stability. Among models receiving varying numbers of dose masks, the configuration with ten masks achieved the highest predictive accuracy.

**Conclusion:**

This study proposes a dose mask-assisted method for lung cancer IMRT dose prediction. It demonstrates high accuracy and robustness in clinical radiotherapy scenarios with various prescription schemes, including conventional radiotherapy and SIB. The inclusion of additional dose masks significantly improved model performance, with prediction accuracy increasing as the number of masks increased.

## 1 Introduction

Lung cancer is one of the leading malignancies globally in terms of both incidence and mortality (1), and radiotherapy is considered an effective and commonly used method for tumor control. Over the past few decades, the development of intensity-modulated radiotherapy (IMRT) has significantly improved the effectiveness of lung cancer radiotherapy (2). Treatment planning systems (TPS) are capable of generating high-quality radiotherapy plans, but physicists must repeatedly fine-tune the dose objectives until the desired dose distribution is achieved. This process is time-consuming and highly dependent on the physicist’s experience and skill, leading to significant variability in plan quality (3).

To address this issue, the research community has focused on automating the treatment planning process to reduce manual intervention and accelerate plan optimization (4). Predicting three-dimensional radiotherapy dose distributions has become a popular research direction. In recent years, deep learning methods, especially convolutional neural networks (CNNs), have shown great potential in medical image processing and dose prediction (5). Many U-Net networks, which take CT images and organ contours as input, have successfully predicted voxel-level 3D dose distributions and are widely used in cancers such as prostate cancer (6–11), head and neck cancer (12–16), and cervical cancer (17–20). Similarly, many studies have focused on lung cancer (21–27) IMRT planning. These studies generally train networks using CT and PTV/OARs structures as input, leading to noticeable dose errors in normal tissue regions far from the PTV.

**Figure 1:**
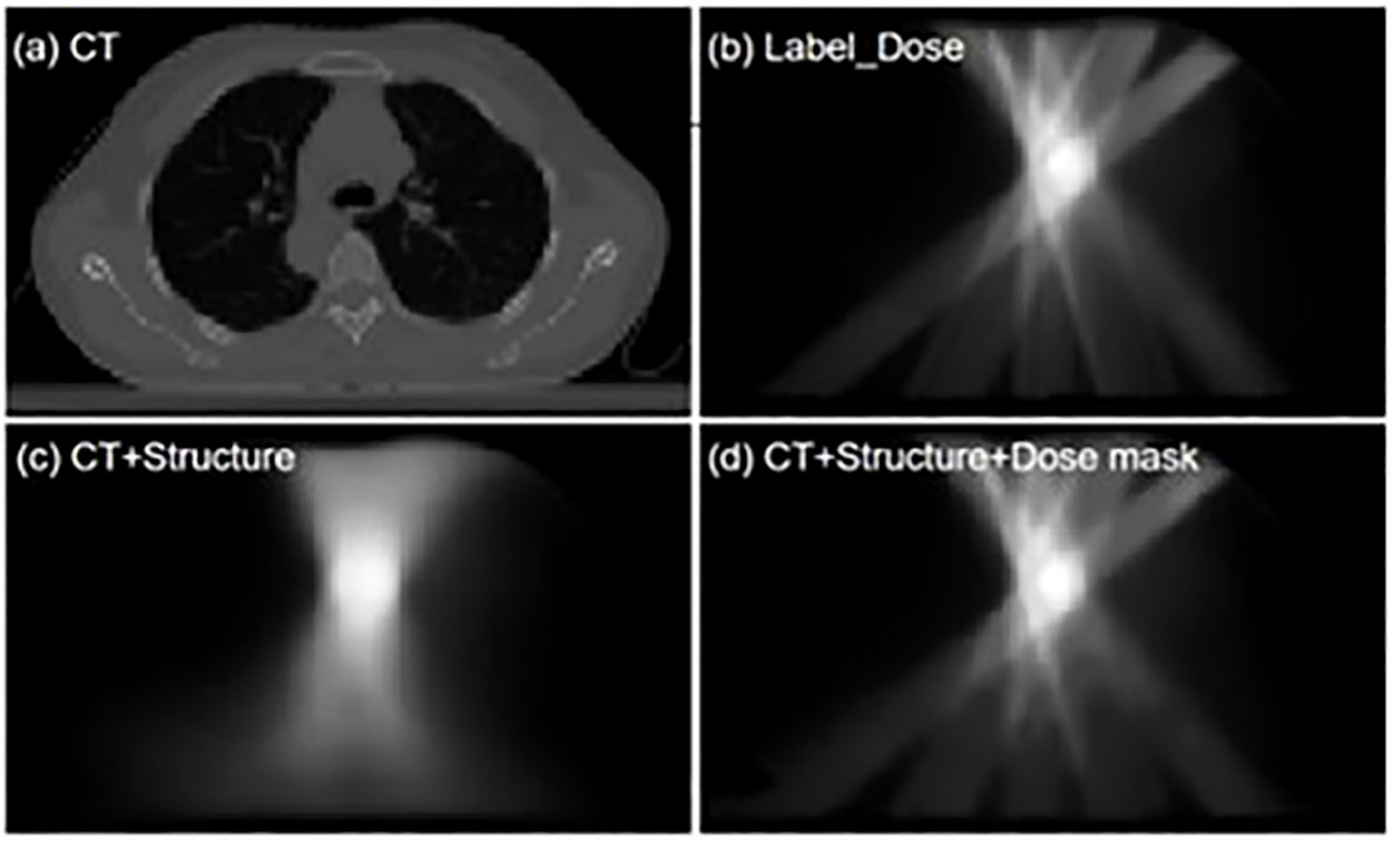
'Visualization comparison of dose distribution predictions between the conventional input model and the dose mask-assisted model. **(a)** CT image of a sample patient; **(b)** Dose distribution of the clinical treatment plan; **(c)** Model-predicted dose distribution with CT and anatomical structures as sole inputs; **(d)** Model-predicted dose distribution with additional dose mask input.

**Figure 1** shows an example of dose errors that may occur using conventional input types. These networks use CT and contour structures as input, resulting in good prediction accuracy at the PTV location where the beams intersect. However, dose errors in normal tissue regions are more pronounced, affecting the dose protection of healthy tissues. Therefore, in dose prediction tasks, it is essential to evaluate not only the target conformity and coverage but also the dose differences along the beam path, with a focus on the protection of organs at risk in the intermediate- and low-dose regions.

To make the model suitable for lung cancer IMRT applications and improve its prediction accuracy and robustness, researchers have adopted complex flux-convolutional wide-beam (FCBB) dose calculation methods (25, 28) to process beam information, enhancing the model’s ability to predict lung cancer IMRT dose distributions for different beam angle setups (21). To further improve the robustness of the model when using a mixed lung cancer dataset with two types of conventional prescription schemes, researchers introduced the Squeeze and Excitation (SE) module, allowing the network to focus more on the dose results for small-volume structures (22, 24). In addition, studies have shown that using cascaded convolutional neural networks can significantly improve both the overall and local dose prediction performance of the model (14).

These methods, through end-to-end learning, minimize reliance on manual features and have shown promising predictive performance in preliminary results. However, existing studies primarily focus on accurately predicting the dose to the planning target volume (PTV), especially when dealing with diverse beam setup strategies, multiple prescription dose schemes, and clinically complex tumor spatial distributions. As a result, they still face significant dose prediction errors in the intermediate- and low-dose regions (22), limiting the accuracy and robustness of the models in different clinical scenarios.

To address these shortcomings, this study introduces the following improvements:

1. Diverse prescription dose schemes and complex tumor spatial distributions: This study incorporates more types of conventional radiotherapy plans and simultaneous integrated boost (SIB) plans (e.g., 60 Gy, 50-60 Gy, 50-60-65 Gy), as well as both unilateral and bilateral tumors, enhancing the model’s applicability and flexibility to cover a wider range of clinical treatment scenarios.
2. Introduction of dose mask information: The model input data includes 10 different dose threshold-based masks, significantly improving prediction accuracy in the intermediate- and low-dose regions and demonstrating the model’s precision in predicting local doses.

By introducing a mixed dataset with multiple prescription schemes and incorporating dose mask information, this study significantly improves the accuracy and generalizability of lung cancer IMRT dose distribution predictions. These improvements not only address the limitations of existing methods in dose prediction but also provide strong technical support for more efficient and personalized treatment planning in clinical practice.

## 2 Materials and methods

### 2.1 Dataset and preprocessing

This dataset includes 190 lung cancer patients who underwent IMRT treatment at our institution up to June 2024 (58 cases of left-sided lung cancer, 88 cases of right-sided lung cancer, and 52 cases of bilateral lung cancer). Ethical approval for the use of patient data was obtained from the institutional review board of our center. The dataset was randomly divided into a training set, validation set, and test set at a ratio of 7:1:2, with 141 cases in the training set, 19 in the validation set, and 35 in the test set. **Table 1** demonstrates the detailed distribution of patients’ prescription regimens. CT images (slice thickness of 3 mm, 512 × 512 matrix) were obtained using a Brilliance CT Big Bore system (Philips Healthcare, Best, the Netherlands). The planning target volume (PTV) and organs at risk (OARs) were contoured by experienced radiation oncologists at our institution. OARs include organs such as the esophagus, heart, lungs, and spinal cord. The planning target volume includes both the conventional planning target volume and the planning gross target volume (PGTV). In all lung cancer IMRT plans, patients receive a dose prescription ranging from 45 to 65 Gy, with each patient having 1 to 3 PTVs.

**Table 1:** Model-specific architectural parameters for the Cascaded U-Net model.

Component	Description	Parameters
Input Layer	Initial 3D volume input (image + dose masks)	Channels: in_ch (e.g. 1 image + N masks), Shape: (X, Y, Z, in_ch)
SingleConv Block	Basic conv unit	Conv3D → InstanceNorm3d → ReLUKernel: 3×3×3, Padding: 1, Stride: variable, Out channels variable
Encoder Stage 1	Two SingleConv at resolution level 1	in_ch → list_ch[1] → list_ch[1], Stride: 1
Encoder Stage 2	Downsample + Two SingleConv at level 2	list_ch[1] → list_ch[2] (stride=2) → list_ch[2] (stride=1)
Encoder Stage 3	Downsample + Two SingleConv at level 3	list_ch[2] → list_ch[3] (stride=2) → list_ch[3] (stride=1)
Encoder Stage 4	Downsample + Two SingleConv at level 4	list_ch[3] → list_ch[4] (stride=2) → list_ch[4] (stride=1)
Encoder Stage 5	Downsample + Two SingleConv at level 5	list_ch[4] → list_ch[5] (stride=2) → list_ch[5] (stride=1)
Decoder UpConv 4	Up-sampling convolution for stage 4	Interpolate ×2, Conv3D(in=list_ch[5], out=list_ch[4]), Kernel: 3×3×3, Padding=1
Decoder Conv 4	Fusion conv at stage 4	SingleConv ×2 on 2×list_ch[4] → list_ch[4]
Decoder UpConv 3	Up-sampling convolution for stage 3	Interpolate ×2, Conv3D(in=list_ch[4], out=list_ch[3]), Kernel: 3×3×3, Padding=1
Decoder Conv 3	Fusion conv at stage 3	SingleConv ×2 on 2×list_ch[3] → list_ch[3]
Decoder UpConv 2	Up-sampling convolution for stage 2	Interpolate ×2, Conv3D(in=list_ch[3], out=list_ch[2]), Kernel: 3×3×3, Padding=1
Decoder Conv 2	Fusion conv at stage 2	SingleConv ×2 on 2×list_ch[2] → list_ch[2]
Decoder UpConv 1	Up-sampling convolution for stage 1	Interpolate ×2, Conv3D(in=list_ch[2], out=list_ch[1]), Kernel: 3×3×3, Padding=1
Decoder Conv 1	Fusion conv at stage 1	SingleConv ×1 on 2×list_ch[1] → list_ch[1]
Output Heads	Final dose prediction outputs	Conv3D(list_ch[1], out_ch=1), Kernel:1×1×1, separate heads for net_A and net_B
Cascade Connection	Two-stage cascaded UNet	net_A output concatenated with input to net_B (in_ch + list_ch_A[1])

All treatment plans were developed for clinical purposes and optimized by experienced physicists at our institution using the Eclipse TPS (Varian Medical Systems, Palo Alto, CA, USA). All plans include 5 to 9 beams and use 6 MV photon energy for irradiation.

The data for each patient includes CT images, anatomical structures, and the planned dose distribution. The resolution of the CT images is 512×512 with a slice thickness of 5 mm. Each PTV and OAR is set as a separate binary mask for input. If a voxel is assigned to an OAR, it is assigned a value of 1 in the corresponding channel; otherwise, it is assigned a value of 0. All CT images, PTV and OAR masks, and dose volumes were resampled to match the pixel size of the dose distribution (1 mm × 1 mm), with the pixel size in the z-axis direction maintained at 5 mm. The CT images, structure masks, and dose distributions were then resampled to a unified grid size (128 × 128 × 128) to reduce computational resource consumption. The CT values were then clipped to the range [-1024, 1500] and normalized to [-1.024, 1.5]. The dose values were normalized to the range [0, 1] based on a standard dose of 65 Gy, which helps the model learn features more effectively. Additionally, data augmentation was applied, including random flips along the X and Z axes, random rotations around the Z axis (0°, 40°, 80°, 120°, 160°, 200°, 240°, 280°, 320°), and random translations with a maximum displacement of 20 pixels. Furthermore, using 65 Gy as the standard prescription dose, dose values were selected at 10% intervals from 10% to 100% of 65 Gy to generate 10 dose thresholds, which were used to generate dose region masks that exceed these thresholds in the dose distribution map.

Our dataset comprises IMRT plans for single-target irradiation and SIB plans for multi-target irradiation, and the cascaded CNN enhances its generalization capability through training. To enable the model to handle variations in different prescription schemes, during data preprocessing, when multiple overlapping PTV structures exist within a patient, the outer PTV structure will crop and discard the inner PTV structure. Then, using 65 Gy as the standard dose, all the trimmed PTVs are merged using the following Equation 1, while assigning the corresponding prescription dose labels. Here, PTV45cut, PTV50cut, PTV60cut, and PTV65cut represent the structures obtained by trimming the planning target volumes prescribed to receive 45 Gy, 50 Gy, 60 Gy, and 65 Gy, respectively.


(1) $$\begin{array}{c} PTVs=\frac{45}{65}\times PTV45_{cut}+\frac{50}{65}\times PTV50_{cut}+\frac{60}{65}\times PTV60_{cut}\\ +\frac{65}{65}\times PTV65_{cut} \end{array}$$

Specifically, each PTV is initially a three-dimensional structure mask where every voxel is assigned a value of 1. Using the following formula and taking 65 Gy as the standard dose, all the trimmed PTV45cut, PTV50cut, PTV60cut, and PTV65cut for a single patient are merged into a single structure, PTVs, with label values ranging between 0 and 1, which serves as the single-channel input for the deep learning model. At the same time, during preprocessing, the dose values in the dose distribution are also scaled using 65 Gy, so that the dose values fall within the range [0, 1] and correspond to the PTV label values. This helps the model understand the dose delivered to the PTV under different prescription schemes, accelerates the convergence of model training, and improves its prediction performance.

### 2.2 Network architecture

#### 2.2.1 Architecture

**Figure 2:**
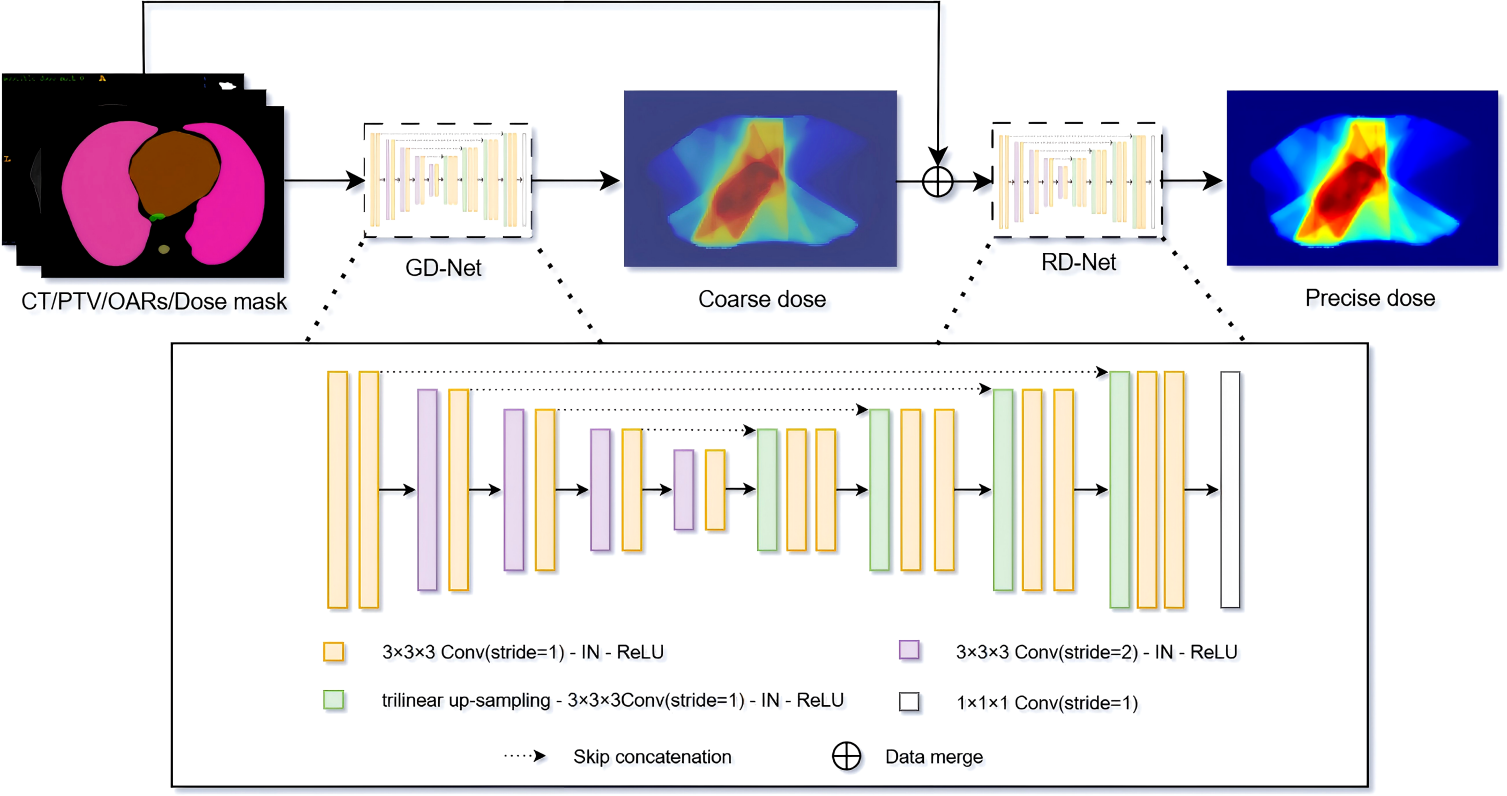
Schematic of the CascU-Net cascaded model for predicting 3D dose distributions. GD-Net and RD-Net are two sequentially connected dose prediction submodels.

In this study, we propose the Cascaded U-Net (CascU-Net) model. The model consists of two cascaded U-Net structures, with the first stage being Global DoseNet (GD-Net) and the second stage being Refine DoseNet (RD-Net), as shown in **Figure 2**. The input channels consist of 1 PTV mask, 7 OAR masks, and 1 CT image, totaling 9 independent input channels. In the encoder of GD-Net, there are 5 resolution levels. Each level extracts key features and reduces image resolution through convolution and downsampling operations. The first level consists of two 3 × 3 × 3 convolutions with a stride of 1 for feature learning; the next 4 levels use 3 × 3 × 3 convolutions (with a stride of 2 for downsampling) and 3 × 3 × 3 convolutions with stride 1 to further extract features. After each downsampling, the number of channels in the feature map is doubled while the spatial dimensions are halved. Thus, the number of channels in the feature map increases from 16 to 256, while the spatial dimensions decrease from 16 × 128 × 128 to 2 × 16 × 16.

In the decoding path of GD-Net, upsampling, convolution, and skip connections are used to restore image details and spatial dimensions. Each decoding level uses trilinear interpolation for upsampling, followed by convolution operations. Each level contains two 3 × 3 × 3 convolutions with a stride of 1, with the last layer containing only one 3 × 3 × 3 convolution and one 1 × 1 × 1 convolution. Skip connections are used to pass the corresponding feature maps from the encoding path to the decoding path to recover information lost during downsampling.

RD-Net receives the output of GD-Net (low-precision dose distribution) along with the original 9 input channels. RD-Net also contains 5 resolution levels in the encoding path and 2 decoding paths, continuing to extract features and ultimately outputting a high-precision dose distribution. Instance normalization and ReLU activation functions are applied to each convolutional layer to prevent overfitting and gradient explosion. Finally, the decoder of RD-Net outputs 1 channel with dimensions restored to 32 × 128 × 128. Detailed model architecture parameter information is shown in **Table 2**.

**Table 2:** Training configuration table.

Setting	Value
Batch Size	2
List of GPU IDs	[0, 1]
Max Iterations	80,000
Learning Rate	3e-4
Weight Decay	1e-4
Loss Function	Custom Loss Function (Loss class)
Training Loss Threshold	0.01
Learning Rate Scheduler	Cosine annealing
Scheduler Arguments	T_max=80,000, eta_min=1e-7, last_epoch=-1
Optimizer	Adam
Train Batch Size	2
Validation Batch Size	2
Training Iterations per Epoch	500
Validation Iterations per Epoch	1
Network Architecture	CascU-Net (Model class) with in_ch=18, out_ch=1, list_ch_A=[-1, 16, 32, 64, 128, 256], list_ch_B=[-1, 32, 64, 128, 256, 512]

#### 2.2.2 Model training

The model uses the mean absolute error (MAE) between the predicted dose and TPS calculated results as the loss function. GD-Net and RD-Net use the same loss function, but since the output of GD-Net is dose distribution D_A_, and RD-Net further improves the prediction accuracy based on GD-Net, outputting dose distribution DB. To train these two sub-networks more effectively, a custom L1 loss function was defined, and its calculation method is shown in Equation 2:


(2) $$\text{L}=\text{α}\sum_{\text{i}=1}^{\text{N}}\frac{\left|{\text{D}}_{\text{A}}\left(\text{i}\right)-\text{GT}\left(\text{i}\right)\right|}{\text{N}}-\text{β}\sum_{\text{i}=1}^{\text{N}}\frac{\left|{\text{D}}_{\text{B}}\left(\text{i}\right)-\text{GT}\left(\text{i}\right)\right|}{\text{N}}\;$$

Here, D_A_(i) represents the predicted dose value of the i-th voxel by GD-Net, D_B_(i) represents the predicted dose value of the i-th voxel by RD-Net, GT(i) is the optimal dose value of the i-th voxel, and N is the total number of voxels that can receive dose. Considering the relationship between GD-Net and RD-Net, as well as the importance of RD-Net in the final dose distribution prediction, α and β are set to 0.5 and 1, respectively.

The network is trained using the cascaded U-Net on a workstation equipped with two 24GB Nvidia RTX 3090 GPUs. The model uses Kaiming initialization[34] for weight initialization, with a batch size of 2, a maximum of 80,000 iterations, 68 iterations per epoch, for a total of 1,176 epochs. The Adam optimizer is used to accelerate convergence and improve training efficiency. The initial learning rate is set to 3e-4, and a cosine annealing strategy is used to gradually reduce the learning rate at each epoch until the minimum learning rate (1e-7) is reached, at which point training stops. **Table 3** shows the detailed training configuration information.

**Table 3:** Distribution of patient prescription dose types.

Sublet	50Gy	60Gy	45-60Gy	50-60Gy	50-65Gy	50-60-65Gy	Sum
Training set	11	47	8	48	5	17	136
Validation set	2	6	1	7	1	2	19
Test set	3	10	2	13	2	5	35
Sum	16	63	11	68	8	24	190

### 2.3 Experimental grouping

#### 2.3.1 Control group

The first experiment, uses CT images, PTV, bilateral lungs, left lung, right lung, spinal cord, esophagus, and heart as input to the neural network. An independent dataset is used for training and evaluation, with the goal of observing the dose distribution prediction results based solely on CT, PTV, and OARs.

#### 2.3.2 Comparative experiments

The first experiment uses only CT, PTV masks, and OARs masks as inputs. While these inputs aid the model in effectively predicting the dose distribution, they lack the information necessary to help the model learn the rate and direction of dose falloff in regions distant from the PTV. Therefore, incorporating different numbers of dose masks to improve dose attenuation in the low and intermediate dose regions is a valuable regions of research. In this study, five input combinations were set up and five models were trained using the same patient dataset:

1. CT + PTV + OARs + BODY mask.
2. CT + PTV + OARs + BODY mask + 3 dose masks (masks corresponding to doses greater than 65 Gy at 10%, 50%, and 90%).
3. CT + PTV + OARs + BODY + 5 dose masks (masks corresponding to doses greater than 65 Gy at 10%, 30%, 50%, 70%, and 90%).
4. CT + PTV + OARs + BODY + 7 dose masks (masks corresponding to doses greater than 65 Gy at 10%, 20%, 30%, 50%, 60%, 70%, and 90%).
5. CT + PTV + OARs + BODY + 10 dose masks (masks corresponding to doses greater than 65 Gy at 10%, 20%, 30%, 40%, 50%, 60%, 70%, 80%, 90%, and 100%).

**Figure 3:**
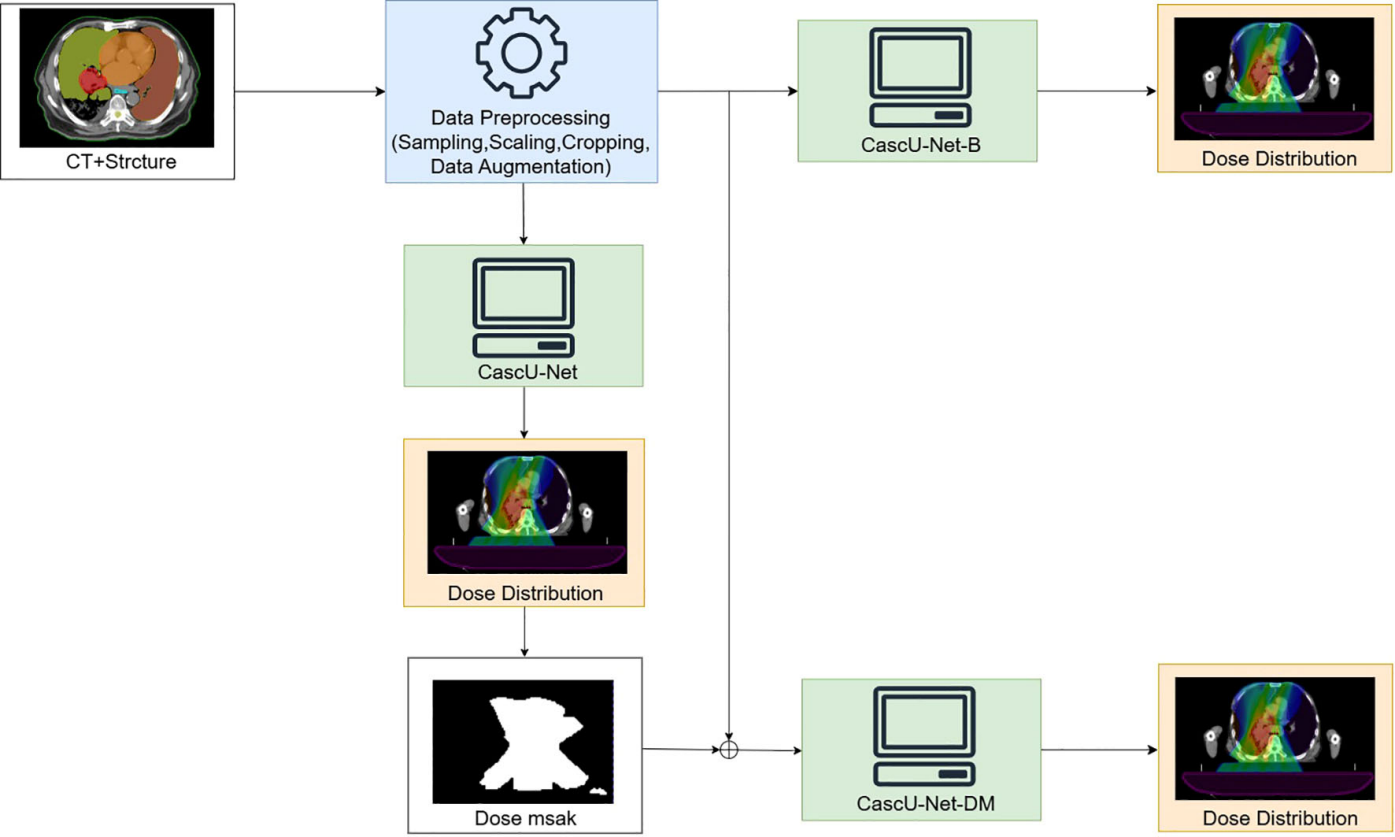
Overview of the data preprocessing workflow and the training of a 3D network to produce voxel-based dose distributions in 3D.

The model in the first group uses only CT, PTV masks, and OAR masks as inputs, referred to as CascU-Net-B (Basic Cascaded U-Net). Models in groups b-e use CT, PTV mask, OARs mask, and dose masks as inputs, referred to as CascU-Net-DM (Dose Mask-Assisted Cascaded U-Net). Furthermore, to make the CascU-Net-DM model applicable to clinical radiotherapy scenarios, we pre-trained a dose distribution prediction model, CascU-Net, which has the same structure as shown in **Figure 3**. Each dose mask was generated by threshold segmentation of the dose distribution results predicted by the CascU-Net model, thereby preventing issues where new patients might be unable to use the model due to the absence of dose masks. The detailed workflow is illustrated in **Figure 3**.

### 2.4 Evaluation

The model’s prediction results and the manual planning results are evaluated using the Homogeneity Index (HI), Conformity Index (CI), Mean Absolute Error (MAE), and Dose-Volume Histogram (DVH).

HI is used to evaluate the uniformity of the PTV dose distribution, defined as in Equation 3. Here, D_n_ represents the dose received by n% of the volume, and D_p_ represents the prescribed dose.


(3) $$\text{HI}=\frac{{\text{D}}_{2}-{\text{D}}_{98}}{{\text{D}}_{\text{P}}}$$

CI is an important metric for evaluating the dose coverage of radiotherapy treatment plans, as defined in Equation 4. Here, V_T,ref_ represents the PTV volume covered by the prescribed dose, V_T_ represents the PTV volume, and _Vref_ represents the volume covered by the prescribed dose.


(4) $$\text{CI}=\frac{{\text{V}}_{\text{T},\text{ref}}}{{\text{V}}_{\text{T}}}-\frac{{\text{V}}_{\text{T},\text{ref}}}{{\text{V}}_{\text{ref}}}\;\;$$

MAE represents the mean absolute error in the dose within the PTV or OARs between the predicted dose and the manually planned dose, as defined in Equation 5. Here, N represents the number of voxels in the PTV or OARs, D_Pre_(i) represents the predicted dose for voxel i, and D_GT_(i) represents the manually planned dose for voxel i.


(5) $$\text{MAE}=\frac{\sum_{\text{i}=1}^{\text{N}}\left(\left|{\text{D}}_{\text{Pre}}\left(\text{i}\right)-{\text{D}}_{\text{GT}}\left(\text{i}\right)\right|\right)}{\text{N}}$$

To further assess the similarity between the closed isodose curve regions in the model-predicted dose distribution and the clinical results, the study employed the Dice Similarity Coefficient (DSC) as a metric, calculated as shown in Equation 6. Here, A represents the 3D voxel dose volume predicted by the model, while B represents the 3D voxel dose volume from the clinical results. Dose values were selected at 0.5 Gy intervals from 0 Gy to 65 Gy for calculation, and the DSC curve was plotted for evaluation.


(6) $$DSC=\frac{2\left(A\cap B\right)}{A+B}$$

Key dosimetric parameters such as D_99%_, D_98%_, D_95%_, D_max_, D_mean_ for PTV, and D_max_, D_mean_, V_40Gy_, V_30Gy_, V_20Gy_, and V_5Gy_ for OARs were assessed, and the differences and standard deviations between the predicted and manually planned results were calculated. The smaller the mean difference and standard deviation, the higher the accuracy of the prediction results.

This study employs statistical tests (paired t-test or Wilcoxon signed-rank test, depending on the normality assumption of the differences) to evaluate the final predictive efficacy. All tests were conducted at a significance level of α = 0.05, with p< 0.05 indicating that the dose differences are statistically significant.

## 3 Results

### 3.1 Impact of dose masks

**Tables 4** and **5** summarize the mean absolute errors (MAE) of the PTV and OARs dosimetric parameters for 35 patients. In CascU-Net-DM, the MAEs of nearly all PTV and OARs clinical parameters are significantly reduced compared to CascU-Net-B. With the exception of D2% and Dmax for all PTVs, the MAEs for most structures are below 2%.

**Table 4:** Comparison of PTV clinical dosimetric parameters in CascU-Net-B and CascU-Net-DM through the MAE (mean ± standard deviation).

Evaluation Metrics		Ground truth	CT/PTV/OARs			CT/PTV/OARs/10mask		
			Prediction	MAE	P	Prediction	MAE	P
PTV45	D_2%_ (Gy)	64.89 ± 0.18	61.45 ± 1.98	4.44 ± 3.17	p=0.5	66.12 ± 1.47	2.32 ± 0.20	p=1
	D_95% _(Gy)	44.94 ± 0.06	47.15 ± 0.24	2.21 ± 0.18	p=0.5	44.53 ± 0.01	1.56 ± 0.01	p=1
	D_98%_ (Gy)	43.03 ± 1.93	44.81 ± 0.35	4.28 ± 2.08	p=0.5	42.57 ± 1.73	2.89 ± 0.15	p=1
	D_mean_ (Gy)	54.61 ± 0.69	53.85 ± 1.57	0.88 ± 0.75	p=1	55.1 ± 1.29	0.31 ± 0	p=1
	D_max_ (Gy)	68.74 ± 0.08	63.93 ± 0.63	6.81 ± 4.27	p=0.5	69.11 ± 0.95	4.36 ± 1.62	p=1
	CI	0.32 ± 0.02	0.41 ± 0.10	0.09 ± 0.08	p=0.5	0.33 ± 0.03	0.06 ± 0.05	p=1
	HI	0.41 ± 0.02	0.30 ± 0.01	0.16 ± 0.10	p=0.5	0.5 ± 0.01	0.1 ± 0.05	p=1
PTV50	D_2% _(Gy)	62.93 ± 4.09	62.60 ± 3.01	1.65 ± 1.24	p=0.3	62.52 ± 3.34	1.23 ± 0.76	p=0.9
	D_95% _(Gy)	48.65 ± 1.18	49.68 ± 1.01	1.68 ± 1.63	p<0.05	48.53 ± 1.29	1.22 ± 1.02	0.01
	D_98%_ (Gy)	46.24 ± 1.43	47.19 ± 1.24	1.86 ± 2.05	p<0.05	46.39 ± 1.74	1.35 ± 0.86	p=0.17
	D_mean _(Gy)	54.85 ± 1.76	56.38 ± 1.20	1.58 ± 0.99	p<0.05	54.82 ± 1.93	1.02 ± 0.57	0
	D_max_ (Gy)	65.36 ± 4.69	64.76 ± 3.80	3.75 ± 1.95	p=0.33	64.27 ± 3.81	1.49 ± 1.26	0
	CI	0.40 ± 0.14	0.44 ± 0.13	0.06 ± 0.05	p<0.05	0.35 ± 0.1	0.04 ± 0.03	0.05
	HI	0.30 ± 0.09	0.27 ± 0.05	0.06 ± 0.05	p<0.05	0.3 ± 0.07	0.03 ± 0.03	p=0.59
PTV60	D_2%_ (Gy)	65.79 ± 1.90	65.31 ± 2.07	3.32 ± 4.51	p=0.42	65.72 ± 1.68	1.67 ± 1.15	p=0.94
	D_95%_ (Gy)	58.89 ± 0.86	58.35 ± 1.08	1.38 ± 0.96	0	58.89 ± 1.41	1.15 ± 0.70	p<0.05
	D_98%_ (Gy)	57.29 ± 1.36	56.84 ± 1.84	1.62 ± 1.1	p=0.08	56.91 ± 1.7	1.16 ± 0.87	p<0.05
	D_mean_ (Gy)	62.55 ± 1.20	62.09 ± 1.13	1.29 ± 1.2	p=0.06	62.98 ± 1.21	1.01 ± 0.71	p=0.92
	D_max_ (Gy)	67.01 ± 2.09	66.23 ± 2.27	3.65 ± 4.71	p=0.18	66.67 ± 1.85	1.95 ± 1.43	p<0.05
	CI	0.63 ± 0.16	0.65 ± 0.18	0.11 ± 0.08	p=0.32	0.63 ± 0.16	0.06 ± 0.04	p=0.97
	HI	0.13 ± 0.04	0.13 ± 0.04	0.06 ± 0.08	p=0.97	0.14 ± 0.04	0.04 ± 0.02	p=0.14
PTV65	D_2%_ (Gy)	70.23 ± 1.94	70.66 ± 2.58	6.43 ± 5.47	p=0.39	66.33 ± 1.91	3.09 ± 1.93	p<0.05
	D_95%_ (Gy)	64.07 ± 0.64	64.04 ± 0.86	1.91 ± 1.28	p=0.63	62.56 ± 1.13	0.91 ± 0.17	p=0.58
	D_98%_ (Gy)	62.62 ± 1.19	62.36 ± 1.60	3.18 ± 3.8	p=0.67	61.82 ± 0.94	2.28 ± 1.71	p=0.79
	D_mean_ (Gy)	67.53 ± 1.01	66.87 ± 0.97	1.85 ± 1.04	p=0.45	64.61 ± 1.61	1.31 ± 0.54	p<0.05
	D_max_ (Gy)	71.04 ± 2.12	71.97 ± 2.77	7.68 ± 6.40	p=0.37	66.71 ± 2.01	3.58 ± 1.67	p<0.05
	CI	0.70 ± 0.08	0.69 ± 0.08	0.24 ± 0.17	p=0.64	0.42 ± 0.21	0.17 ± 0.07	p=0.19
	HI	0.13 ± 0.08	0.15 ± 0.07	0.12 ± 0.10	p=0.59	0.09 ± 0.04	0.06 ± 0.07	p=0.13

**Table 5:** Comparison of OARs clinical dosimetric parameters in CascU-Net-B and CascU-Net-DM through the MAE (mean ± standard deviation).

Evaluation Metrics	Ground truth	CT/PTV/OARs			CT/PTV/OARs/10mask		
		Prediction	MAE	P	Prediction	MAE	P
Lung_L V_5Gy_ (%)	41.07 ± 26.62	41.17 ± 27.33	5.08 ± 5.05	p=0.79	40.66 ± 26.6	3.25 ± 3.21	p=0.36
Lung_L V_20Gy_ (%)	21.23 ± 23.10	21.24 ± 24.18	2.57 ± 2.57	p=0.53	21.42 ± 23.2	1.72 ± 1.99	p=0.59
Lung_L D_mean_ (Gy)	12.37 ± 12.32	12.36 ± 12.68	0.85 ± 0.73	p=0.75	12.34 ± 12.39	0.73 ± 0.61	p=0.23
Lung_R V_5Gy_ (%)	44.90 ± 22.76	45.45 ± 21.19	3.75 ± 3.55	p=0.75	44.58 ± 22.74	2.96 ± 2.37	p=0.85
Lung_R V_20Gy_ (%)	23.56 ± 19.58	23.21 ± 19.60	2.21 ± 1.61	p=0.66	23.82 ± 19.63	1.58 ± 1.14	p=0.45
Lung_R D_mean_ (Gy)	13.34 ± 10.76	13.25 ± 10.63	0.84 ± 0.60	p=0.64	13.36 ± 10.84	0.53 ± 0.41	p=0.55
Double Lung V_5Gy_ (%)	41.14 ± 14.61	41.10 ± 13.97	3.98 ± 3.06	p=0.87	40.75 ± 14.67	2.46 ± 2.12	p=0.54
Double Lung V_20Gy_ (%)	20.30 ± 14.91	20.06 ± 14.96	1.53 ± 1.14	p=0.65	20.51 ± 14.89	1.13 ± 0.83	p=0.45
Double Lung D_mean_ (Gy)	11.80 ± 9.21	11.72 ± 9.25	0.65 ± 0.38	p=0.39	11.8 ± 9.28	0.38 ± 0.29	p=0.89
Heart V_30Gy _(%)	13.51 ± 18.89	13.90 ± 18.78	1.72 ± 2.55	p=0.11	13.32 ± 18.86	1.11 ± 1.22	p=0.5
Heart V_40Gy_ (%)	9.66 ± 17.62	9.65 ± 17.51	1.64 ± 1.97	p=0.58	9.83 ± 17.67	1.19 ± 1.49	p=0.24
Heart D_mean_ (Gy)	10.56 ± 11.14	10.82 ± 11.13	0.96 ± 1.03	p=0.6	10.58 ± 11.2	0.65 ± 0.58	p=0.43
Esophagus D_max_ (Gy)	53.18 ± 14.13	53.30 ± 14.30	3.44 ± 4.09	p=0.7	53.22 ± 14.29	2.97 ± 2.18	p=0.97
Esophagus D_mean_ (Gy)	20.37 ± 11.97	20.51 ± 12.09	1.36 ± 1.13	p=0.18	20.38 ± 12	0.82 ± 0.76	p=0.83
Spinalcord D_max_ (Gy)	37.89 ± 7.37	37.28 ± 8.30	4.04 ± 4.63	p=0.36	38.14 ± 7.5	3.66 ± 3.38	p=0.15

**Figure 4:**
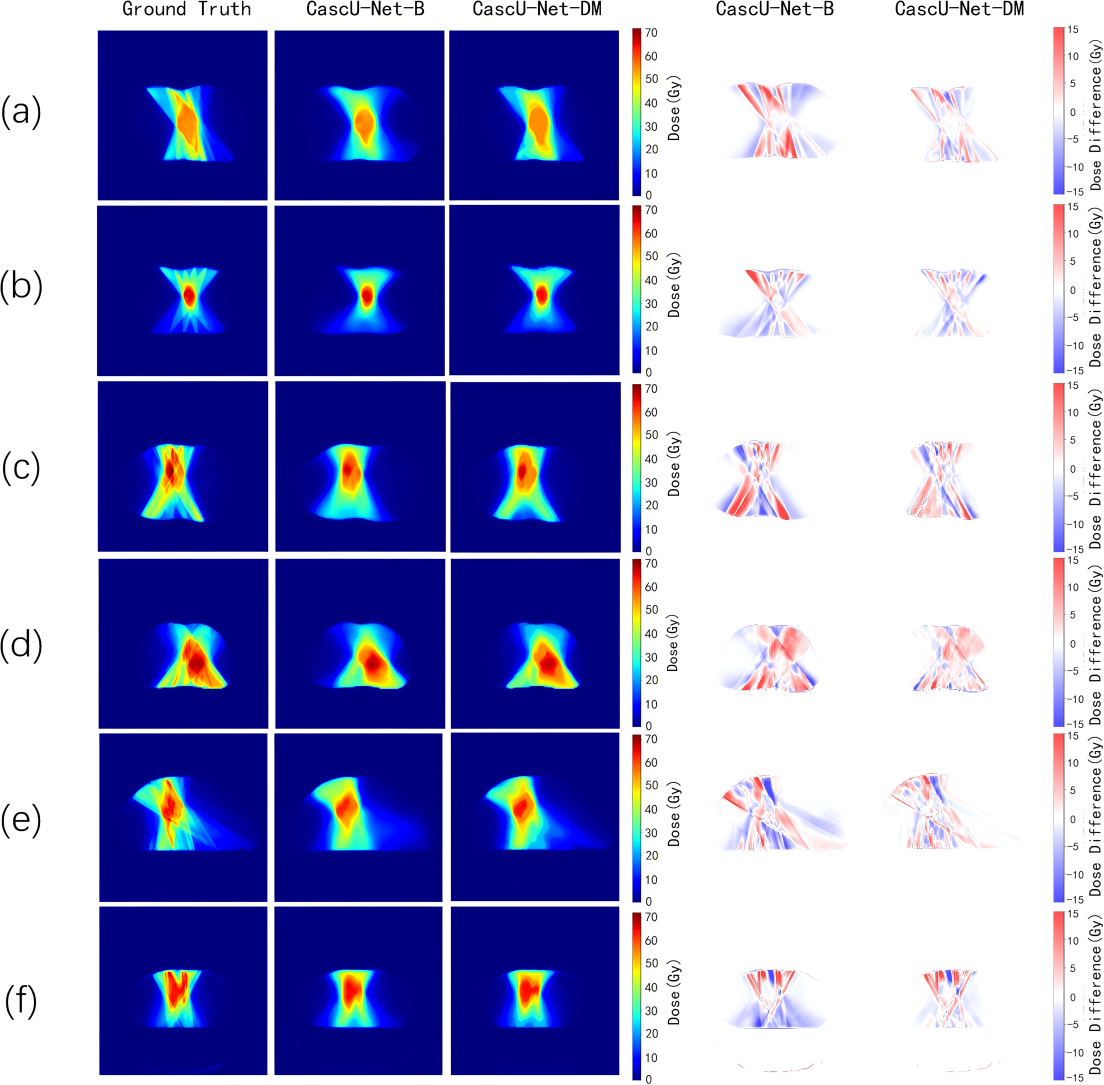
The ground-truth, predicted dose results from different models, and the dose differences between the predicted results and the ground-truth for representative patients from six different prescription schemes in the test set are presented. The corresponding six prescription schemes are as follows: **(a)** 50 Gy; **(b)** 60 Gy; **(c)** 50-65 Gy; **(d)** 50-60-65 Gy; **(e)** 50-60 Gy; **(f)** 45-60 Gy.

**Figure 5:**
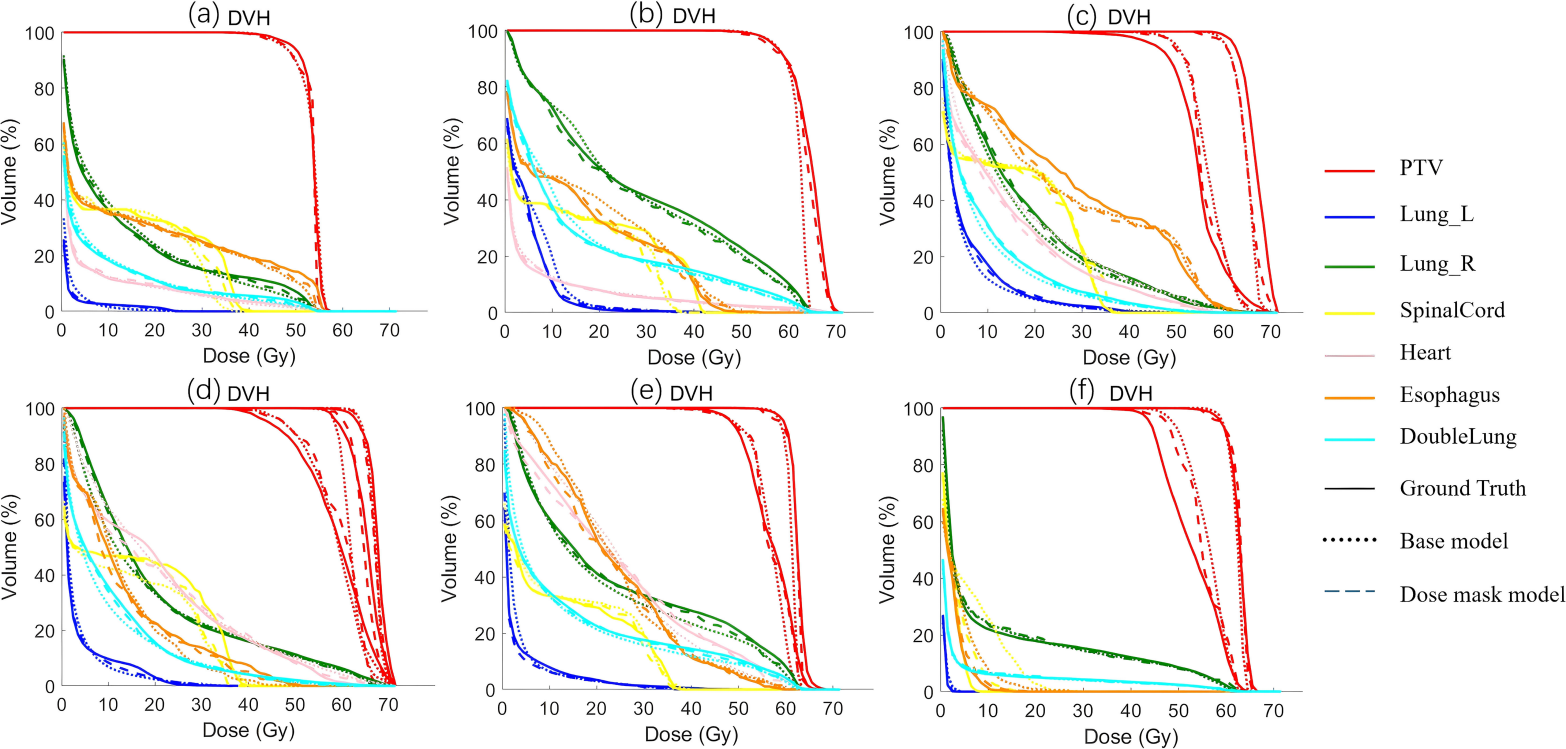
The DVH of the predicted results from different models and the ground-truth for representative patients from six different prescription schemes in the test set are presented. The corresponding six prescription schemes are as follows: **(a)** 50 Gy; **(b)** 60 Gy; **(c)** 50-65 Gy; **(d)** 50-60-65 Gy; **(e)** 50-60 Gy; **(f)** 45-60 Gy.

**Figure 4** shows the dose distribution of CascU-Net-B and CascU-Net-DM on the cross-sectional images of six patients in the test set, including clinical dose distributions, predicted dose distributions, and dose difference maps. Six patients were randomly selected from the test set of 6 prescribed dose, corresponding to the following prescribed dose: (a) 50 Gy; (b) 60 Gy; (c) 50-65 Gy; (d) 50-60-65 Gy; (e) 50-60 Gy; (f) 45-60 Gy. In the dose distribution plots for all prescriptions, the difference between the predicted voxel dose and the clinical outcome was much smaller for CascU-Net-DM.The protective effect of CascU-Net-DM on OARs was comparable to that of the manual plan in the low and medium dose regions, while the dose prediction accuracy improvement effect was limited in the higher dose regions. **Figure 5** shows the dose-volume histograms (DVH) of the clinical dose distributions versus the predicted dose distributions for all the patients in **Figure 4** in turn. From the DVH plot, it can be seen that the PTV and OARs prediction results of CascU-Net-DM are highly consistent with the clinical results, realizing the dose coverage ability of the target area and the protection of the critical organs comparable to the clinical results, and especially in the low and intermediate dose regions, the error is significantly reduced.

### 3.2 Ablation experiment

**Tables 6** and **7** present the mean absolute error (MAE) of the dose evaluation metrics for PTV and OARs using CascU-Net-DM with four different numbers of dose masks as inputs. As the number of dose masks increases, the overall clinical evaluation metric difference for PTV and OARs are significantly reduced, with the network using 10 dose masks performing the best.

**Table 6:** Comparison of PTV clinical dosimetric parameters in the ablation experiment for 35 test set patients using CascU-Net-DM with different numbers of dose masks through the MAE (mean ± standard deviation).

Evaluation Metrics		3 mask		5 mask		7 mask		10 mask	
		MAE	p-value	MAE	p-value	MAE	p-value	MAE	p-value
PTV45	D_2%_ (Gy)	3.75 ± 0.48	p=1	3.39 ± 2.19	p=1	2.45 ± 2.38	p=0.5	2.32 ± 0.20	p=1
	D_95%_ (Gy)	1.92 ± 1.37	p=0.5	1.72 ± 0.10	p=1	1.61 ± 0.21	p=1	1.56 ± 0.01	p=1
	D_98%_ (Gy)	3.55 ± 2.37	p=1	3.02 ± 1.01	p=1	2.92 ± 0.91	p=1	2.89 ± 0.15	p=1
	D_mean_ (Gy)	0.59 ± 0.41	p=1	0.57 ± 0.41	p=1	0.32 ± 0.17	p=0.5	0.31 ± 0	p=1
	D_max_ (Gy)	5.33 ± 3.10	p=1	4.9 ± 3.53	p=1	4.87 ± 2.95	p=0.5	4.36 ± 1.62	p=1
	CI	0.08 ± 0.07	p=0.5	0.07 ± 0.05	p=1	0.07 ± 0.05	p=1	0.06 ± 0.05	p=1
	HI	0.13 ± 0.03	p=1	0.11 ± 0.06	p=1	0.11 ± 0.06	p=1	0.1 ± 0.05	p=1
PTV50	D_2%_ (Gy)	1.49 ± 1.08	p=0.49	1.41 ± 0.87	p=0.4	1.37 ± 1.04	p=0.84	1.23 ± 0.76	p=0.9
	D_95%_ (Gy)	1.53 ± 1.02	p=0.09	1.4 ± 1.11	p<0.05	1.32 ± 0.81	p<0.05	1.22 ± 1.02	p<0.05
	D_98%_ (Gy)	1.75 ± 1.06	p=0.4	1.69 ± 0.97	p=0.29	1.51 ± 0.95	p=0.15	1.35 ± 0.86	p=0.17
	D_mean_ (Gy)	1.28 ± 1.07	p<0.05	1.22 ± 1.14	p<0.05	1.1 ± 0.8	p<0.05	1.02 ± 0.57	p<0.05
	D_max_ (Gy)	1.86 ± 1.38	p<0.05	1.74 ± 0.95	p=0.23	1.54 ± 0.9	p<0.05	1.49 ± 1.26	p<0.05
	CI	0.06 ± 0.04	p<0.05	0.05 ± 0.03	p=0.92	0.04 ± 0.03	p=0.64	0.04 ± 0.03	0.05
	HI	0.04 ± 0.04	p=0.42	0.04 ± 0.02	p=0.67	0.04 ± 0.02	p=0.49	0.03 ± 0.03	p=0.59
PTV60	D_2%_ (Gy)	2.05 ± 1.70	p=0.35	1.95 ± 1.63	p=0.68	1.81 ± 1.56	p<0.05	1.67 ± 1.15	p=0.94
	D_95%_ (Gy)	1.35 ± 0.99	p<0.05	1.24 ± 0.88	p<0.05	1.27 ± 0.79	p=0.05	1.15 ± 0.70	p<0.05
	D_98%_ (Gy)	1.51 ± 0.79	p<0.05	1.42 ± 0.75	p<0.05	1.36 ± 1.01	p<0.05	1.16 ± 0.87	p<0.05
	D_mean_ (Gy)	1.22 ± 0.74	p=0.08	1.2 ± 0.79	p=0.5	1.16 ± 0.95	p=0.39	1.01 ± 0.71	p=0.92
	D_max_ (Gy)	2.06 ± 2.04	p=0.52	2.03 ± 1.33	p=0.2	2.03 ± 1.29	p<0.05	1.95 ± 1.43	p<0.05
	CI	0.08 ± 0.08	p=0.62	0.07 ± 0.06	p=0.25	0.06 ± 0.05	p=0.66	0.06 ± 0.04	p=0.97
	HI	0.06 ± 0.04	p<0.05	0.05 ± 0.03	p<0.05	0.05 ± 0.04	p=0.12	0.04 ± 0.02	p=0.14
PTV65	D_2%_ (Gy)	3.67 ± 2.34	p<0.05	3.1 ± 1.63	p<0.05	3.1 ± 1.48	p<0.05	3.09 ± 1.93	p<0.05
	D_95%_ (Gy)	1.4 ± 0.74	p=0.73	1.19 ± 0.53	p=0.7	1.07 ± 0.39	p=0.33	0.91 ± 0.17	p=0.58
	D_98%_ (Gy)	2.55 ± 2.48	p=0.77	2.52 ± 1.98	p=0.88	2.41 ± 2.49	p=0.91	2.28 ± 1.71	p=0.79
	D_mean_ (Gy)	1.67 ± 0.83	p<0.05	1.53 ± 1.18	p<0.05	1.46 ± 0.76	p<0.05	1.31 ± 0.54	p<0.05
	D_max_ (Gy)	4.01 ± 2.55	p<0.05	3.91 ± 1.99	p<0.05	3.81 ± 1.4	p<0.05	3.58 ± 1.67	p<0.05
	CI	0.22 ± 0.23	p=0.08	0.2 ± 0.12	p=0.07	0.19 ± 0.08	p<0.05	0.17 ± 0.07	p=0.19
	HI	0.07 ± 0.08	p=0.21	0.07 ± 0.04	p=0.12	0.06 ± 0.03	p=0.02	0.06 ± 0.07	p=0.13

**Table 7:** Comparison of OARs clinical dosimetric parameters in the ablation experiment for 35 test set patients using CascU-Net-DM with different numbers of dose masks through the MAE (mean ± standard deviation).

Evaluation Metrics	3 mask		5 mask		7 mask		10 mask	
	MAE	p-value	MAE	p-value	MAE	p-value	MAE	p-value
Lung_L V_5Gy_ (%)	3.62 ± 3.65	p=0.53	3.5 ± 3.35	p=0.57	3.42 ± 3.52	p=0.5	3.25 ± 3.21	p=0.36
Lung_L V_20Gy_ (%)	2.03 ± 2.42	p=0.06	1.92 ± 2.38	p=0.64	1.82 ± 2.32	p=0.78	1.72 ± 1.99	p=0.59
Lung_L D_mean_ (Gy)	0.81 ± 0.71	p=0.12	0.78 ± 0.68	p=0.2	0.79 ± 0.64	p=0.21	0.73 ± 0.61	p=0.23
Lung_R V_5Gy_ (%)	3.22 ± 2.66	p=0.78	3.04 ± 2.51	p=0.6	3.04 ± 2.36	p=0.96	2.96 ± 2.37	p=0.85
Lung_R V_20Gy_ (%)	2.02 ± 1.68	p=0.49	1.87 ± 1.85	p=0.5	1.77 ± 1.43	p=0.52	1.58 ± 1.14	p=0.45
Lung_R D_mean_ (Gy)	0.73 ± 0.55	p=0.89	0.65 ± 0.61	p=0.51	0.59 ± 0.37	p=0.33	0.53 ± 0.41	p=0.55
Double Lung V_5Gy_ (%)	2.63 ± 2.18	p=0.56	2.5 ± 2.03	p=0.6	2.53 ± 2.09	p=0.42	2.46 ± 2.12	p=0.54
Double Lung V_20Gy_ (%)	1.4 ± 0.92	p=0.46	1.3 ± 0.96	p=0.92	1.2 ± 1.06	p=0.26	1.13 ± 0.83	p=0.45
Double Lung D_mean_ (Gy)	0.57 ± 0.38	p=0.41	0.46 ± 0.35	p=0.48	0.42 ± 0.31	p=0.95	0.38 ± 0.29	p=0.89
Heart V_30Gy_ (%)	1.39 ± 3.03	p=0.58	1.27 ± 2.34	p=0.81	1.18 ± 1.91	p=0.45	1.11 ± 1.22	p=0.5
Heart V_40Gy_ (%)	1.58 ± 2.20	p=0.19	1.49 ± 2.29	p=0.57	1.24 ± 1.55	p=0.27	1.19 ± 1.49	p=0.24
Heart D_mean_ (Gy)	0.8 ± 0.80	p=0.62	0.76 ± 0.79	p=0.65	0.69 ± 0.55	p=0.75	0.65 ± 0.58	p=0.43
Esophagus D_max_ (Gy)	3.34 ± 3.12	p=0.74	3.19 ± 2.61	p=0.42	3.01 ± 2.57	p=0.63	2.97 ± 2.18	p=0.97
Esophagus D_mean_ (Gy)	1.09 ± 0.96	p=0.51	0.88 ± 0.74	p=0.2	0.74 ± 0.57	p=0.67	0.82 ± 0.76	p=0.83
Spinalcord D_max_ (Gy)	3.91 ± 4.45	p=0.62	3.83 ± 4.70	p=0.10	3.76 ± 3.6	p=0.08	3.66 ± 3.38	p=0.15

**Figure 6:**
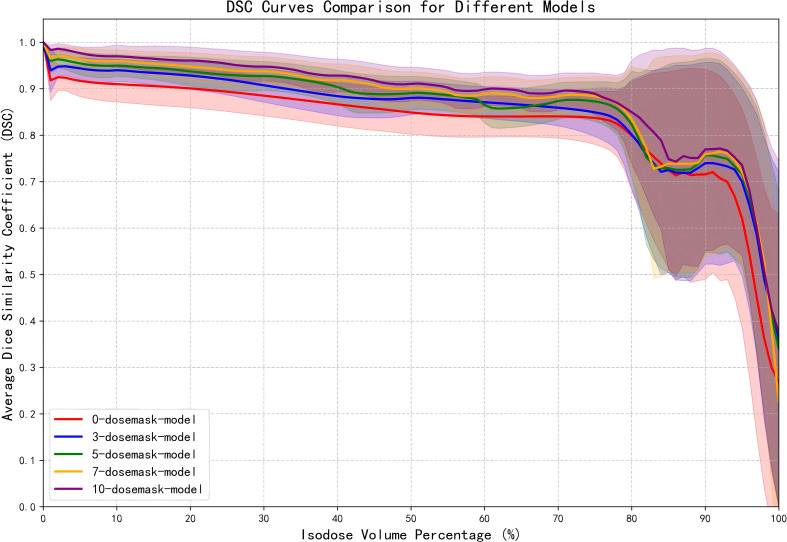
DSC curves of the ablation model at various isodose volumes. The shaded regions represents the standard deviation of DSC values among patients in the test set.

**Figure 6** illustrates the DSC curves of the ablation model across various isodose volumes. For the model trained solely on CT images and organ contours, DSC values remain largely stable between 0.8 and 0.9 for regions below the 80% isodose volume. Upon introducing a limited number of dose masks, predictive accuracy at the 10%, 50%, and 90% isodose volumes increases markedly, with adjacent isodose levels also showing improvement. As additional dose masks are incorporated, the corresponding DSC values steadily converge toward 1. The shaded bands surrounding each curve denote the standard deviation of DSC values across all patients in the test set, indicating that prediction stability is maximized when ten dose masks are employed.

**Figure 7:**
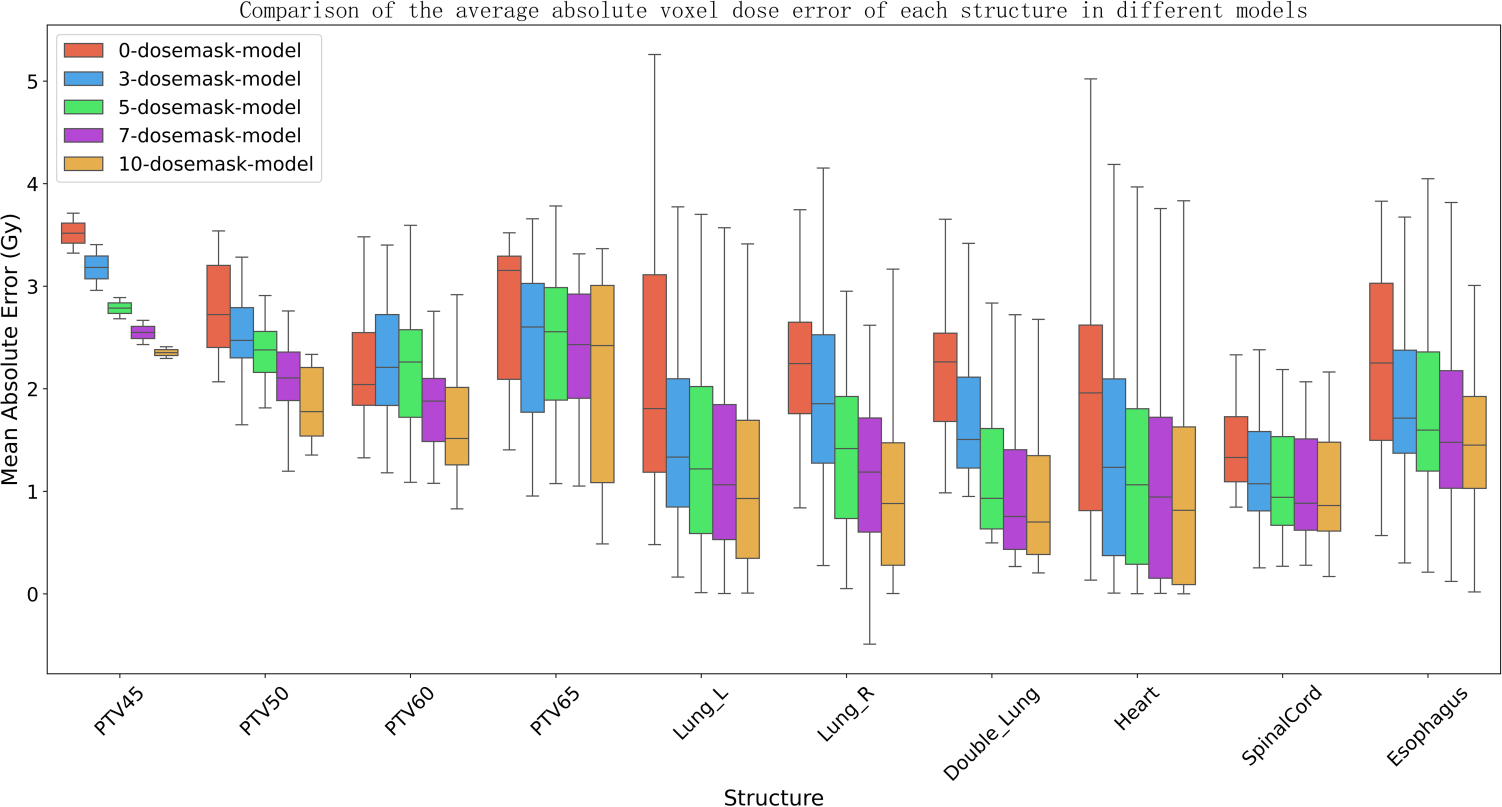
The boxplot depicts the mean absolute error (MAE) of the dose distribution prediction model for different numbers of input dose masks. The box represents the first quartile (Q1) and third quartile (Q3), with the upper and lower whiskers indicating the maximum and minimum values, respectively. The median is shown by the horizontal line within the box, and outliers are marked by white dots.

**Figure 7** presents the voxel‐wise dose MAE for each anatomical structure, displayed as boxplots for models receiving different numbers of dose masks as input. It is apparent that increasing the number of input dose masks yields a uniformly positive effect on dose‐prediction accuracy across all structures. The benefit is most pronounced for larger lung volumes, whereas smaller structures near the tumor—such as the spinal cord and esophagus—exhibit more modest improvements. Furthermore, as the count of dose masks grows, the MAE distributions for all structures become more concentrated and the overall dose error decreases. This suggests that, although adding further masks beyond a certain point offers diminishing returns in mean performance enhancement, it still contributes to greater stability of the predictive results.

**Figure 8:**
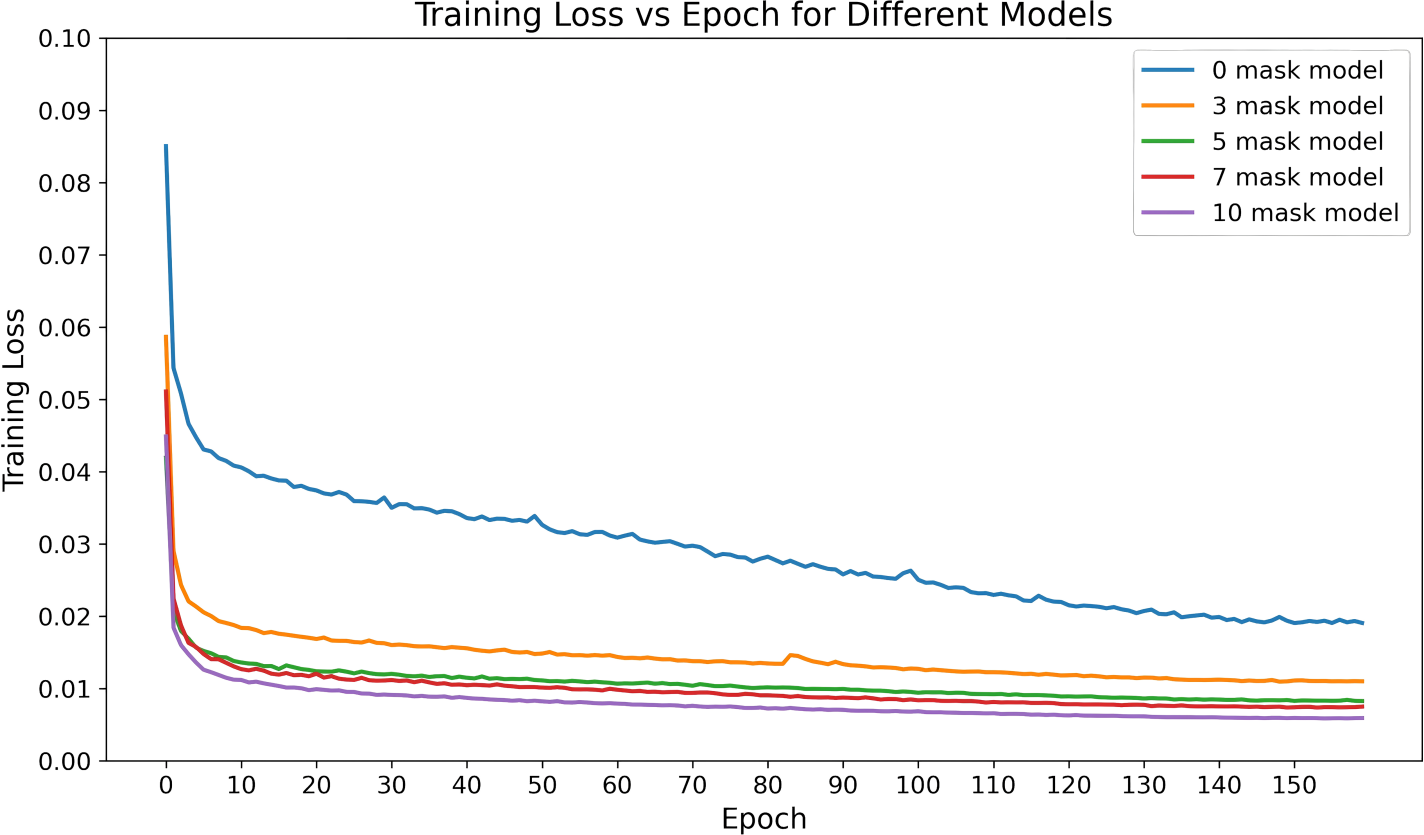
Training loss variation over time for the CascU-Net model with different numbers of dose masks as inputs.

**Figure 8** shows the changes in training loss for the models with different numbers of dose masks as inputs. From the figure, it can be observed that as the number of masks increases, the training loss gradually decreases and stabilizes at a lower value. This indicates that adding dose masks provides additional prior information, helping the model better fit the data and improve training performance. Especially in the 7-mask and 10-mask models, the training loss decreases more rapidly, and the final loss values are lower, suggesting that dose masks provide significant support in these models.

## 4 Discussion

This study uses a cascaded model to predict the multi-prescription dose distributions for lung cancer IMRT. Compared to existing studies on lung cancer IMRT dose distribution prediction, this study’s dataset covers a variety of conventional radiotherapy and simultaneous integrated boost radiotherapy prescription schemes, making it applicable to a broader range of clinical scenarios. Additionally, this study incorporates dose masks as inputs to assist model training, thereby improving the prediction accuracy for the low and intermediate dose regions. For most PTV and OARs metrics, the dose mask-assisted model showed a significant reduction in errors compared to clinical results, demonstrating the effectiveness of using dose masks as additional input information. Moreover, this model has broad applicability across multiple clinical scenarios without the need to train separate models for each prescription scheme.

In the first experiment, the base model was trained and evaluated, followed by the addition of ten dose masks as auxiliary information for further training. CascU-Net-B exhibits larger errors in regions far from the target, whereas CascU-Net-DM significantly reduces these errors in those regions. This may be because dose masks help the model more effectively learn the direction and rate of dose falloff in the low and intermediate dose regions, resulting in more precise dose sculpting in these regions by the improved model. For the D_max_ metric of the PTV, the model incorporating dose masks also showed significant improvement. Although D_max_ is an extreme value with a large range of variation, the stability of the model improved noticeably after adding the masks, indicating that the dose masks help the model better capture dose fluctuations in the clinical plan. Therefore, models incorporating dose mask information are a more effective alternative to models that only input CT and structure delineation.

Compared to CascU-Net-B, the model in this study shows a lower mean absolute error (MAE) after the introduction of dose masks, indicating that the predicted results are numerically closer to the clinical plan. However, based on statistical tests of the predicted and clinical plan values, the p-value shows that there is still a statistically significant difference. The reason is that, the p-value results are closely related to the sample size and the consistency of the bias direction. Even small biases, if consistent in the majority of patients and with a sufficiently large sample size, will be detected by the statistical test and yield p< 0.05. From a clinical application perspective, what matters more is whether the prediction error can be kept within an acceptable range. Although statistical differences still exist, our model reduces the MAE by 1.5%, which provides higher reference value for clinical dose distribution decisions and subsequent plan optimization.

In the second experiment, the impact of different numbers of dose masks on the performance of the dose prediction network was investigated. **Figure 7** shows that as the number of dose masks increases, the model’s prediction accuracy significantly improves, the MAE value gradually decreases, and the prediction results become more stable. This aligns with the study’s hypothesis, as the denser the dose mask intervals, the more the model can learn the relationships between dose masks and dose distributions, including the direction and rate of dose falloff near organs at risk. The overall trend indicates that dose masks are an effective supplement in deep learning models for radiation dose prediction applications. Compared to traditional models, models with varying numbers of masks significantly improved the stability of the training process and ultimately achieved lower training losses. These findings provide important references for further improving the accuracy of deep learning models based on clinical prior information in the future.

This study also has some limitations. Although incorporating dose masks substantially improved model performance—particularly in mid-to-low-dose regions for OARs protection—the predictive gains in high-dose areas were marginal. This shortcoming likely arises because using a fixed number of equally spaced thresholds (e.g., ten masks) yields relatively broad dose intervals per mask. At the steep dose-falloff regions bordering the high-dose volume, such coarse masks cannot capture the submillimeter-scale rapid dose-falloff, leaving the model unable to learn these fine-grained transitions. Furthermore, low-dose regions occupy a much larger volume in the dataset; even with a voxel-wise loss, the network tends to prioritize minimizing global error in the volumetrically dominant zones. Consequently, errors in the relatively sparse high-gradient regions contribute little to the overall loss, and the model achieves limited convergence improvements there. In future work, we plan to introduce a gradient-weighted loss that assigns higher penalty to voxels in steep dose-falloff areas. We will also explore finer thresholding in the high-dose region—such as generating masks every 2 Gy—or adaptive mask generation driven by local gradient magnitude to enhance spatial resolution in these critical zones.

To our knowledge, this is the first dose prediction network that considers dose mask auxiliary information. The information on dose falloff direction and rate in different dose regions is clinically significant as it helps improve dose sculpting in the low and intermediate dose regions, which is of critical importance for protecting important organs at risk. Additionally, it is worth noting that other models (21, 24, 25, 27) only consider common 50Gy, 60Gy, and 50Gy/60Gy conventional radiotherapy schemes, whereas this model considers a mixed dataset of 2 conventional radiotherapy and 4 simultaneous integrated boost radiotherapy prescription dose combinations, making this model more applicable and robust in real clinical radiation therapy scenarios. When the dose distribution generated by the model can successfully be used to create treatment plans in commercial TPS, it will greatly advance the development of one-stop radiation therapy and adaptive radiation therapy, leading to greater clinical benefits for patients.

## 5 Conclusion

This study innovatively proposes a dose prediction method based on the CascU-Net model, which significantly improves the prediction accuracy of lung cancer IMRT dose distribution by incorporating dose masks and effectively addresses the issue of diverse prescription schemes in the dataset. In lung cancer IMRT dose distribution prediction research, multiple conventional radiotherapy and simultaneous integrated boost radiotherapy prescription schemes can be used as mixed data inputs to the model, rather than being limited to a single prescription dataset, thereby avoiding the need to configure multiple models for different radiation therapy scenarios.

## Data Availability

The original contributions presented in the study are included in the article/supplementary material. Further inquiries can be directed to the corresponding authors.
